# Physical Activity in Different Preschool Settings: An Exploratory Study

**DOI:** 10.1155/2014/321701

**Published:** 2014-06-24

**Authors:** Katrin Röttger, Elke Grimminger, Friederike Kreuser, Lorenz Assländer, Albert Gollhofer, Ulrike Korsten-Reck

**Affiliations:** ^1^Institute of Sport and Sport Science, University of Freiburg, Schwarzwaldstraße 175, 79117 Freiburg, Germany; ^2^Faculty of Psychology and Human Movement Science, Institute of Human Movement Science, University of Hamburg, Turmweg 2, 20148 Hamburg, Germany; ^3^Exercise Medicine and Sport, University Medical Center, University of Freiburg, Hugstetter Straße 55, 79106 Freiburg, Germany

## Abstract

*Introduction*. Physical activity (PA) in preschoolers is vital to protect against obesity but is influenced by different early-life factors. The present study investigated the impact of different preschool programs and selected family factors on preschoolers' PA in different countries in an explorative way. *Methods*. The PA of 114 children (age = 5.3 ± 0.65 years) attending different preschool settings in four cities of the trinational Upper Rhine region (Freiburg, Landau/Germany, Basel/Switzerland, and Strasbourg/France) was measured by direct accelerometry. Anthropometrical and family-related data were obtained. Timetables of preschools were analyzed. *Results*. Comparing the preschool settings, children from Strasbourg and Landau were significantly more passive than children from Basel and Freiburg (*P* < .01). With regard to the family context as an important early-life factor, a higher number of children in a family along with the mother's and child's anthropometrical status are predictors of engagement in PA. *Conclusion*. More open preschool systems such as those in Basel, Freiburg, and Landau do not lead to more PA “per se” compared to the highly regimented desk-based system in France. Preliminaries such as special training and the number of caregivers might be necessary elements to enhance PA. In family contexts, targeted PA interventions for special groups should be more focused in the future.

## 1. Introduction

The increasing prevalence of overweight and obese preschoolers represents a challenging public health issue [1]. Early childhood obesity is associated with health consequences that may persist into adolescence and adulthood [2, 3]. Physical activity (PA) is one of the factors that influence the healthy development of children and their weight, but the majority of preschoolers tend to be inactive [4, 5]. Inactivity has been suggested as being one of the key factors contributing to the obesity epidemic in children [6, 7]. In contrast, PA participation in preschool children contributes to motor skill and psychosocial development and is vital for establishing lifelong physical activity habits during the preschool years, which could protect against weight gain later in life [6, 8].

Families with young children have been identified as a particular risk group regarding lower levels of PA of both mother and father (compared to women and men without children) [9]. This might be an important factor for establishing PA habits in young children as children's and parents' PA levels are associated [10] and therefore parents function as a role model for their children [11]. These findings at family level give rise to the question of which other early life structures could contribute to an active lifestyle in preschoolers. Because in European countries nearly 90% of preschool-age children attend some form of preschool, the increasing attendance and considerable time spent in these institutions have generated emerging interest in these settings as an important early-life factor of PA in preschoolers [12]. Consequently, one could conclude that the preschool environment is an ideal institution for PA promotion and obesity prevention [13, 14]. However, a review by Reilly concluded that PA levels are typically very low during out-of-home care, with great variability between the settings [14]. In these settings, unstructured PA during recess and free-play times or structured PA during physical education (PE) classes provides different opportunities to achieve the necessary amount of PA. Eveline et al. [15] showed that preschool children are, on average, engaged for half of the time in sedentary behaviors even in structured PE lessons, whereas Gordon et al. [16] detected that outdoor activity and incorporated unstructured activity had a great effect on moderate to vigorous activity (MVPA). Another study suggests that preschoolers' PA could potentially be increased by shorter bouts of structured PA throughout the preschool day [17].

All these results demonstrate the complex backgrounds of PA behavior in this age group and also show that the specific reasons for low PA levels among preschoolers need to be better understood [7].

To guide the development of PA intervention in preschool settings, it is important to identify structures that promote regular PA [18]. In this context, a study of country- or region-specific preschool programs, along with an evaluation of their effects on PA, could be helpful in identifying the chances and risks associated with the promotion of PA in preschool for obesity prevention.

By comparing four cities (Freiburg and Landau in Germany, Basel in Switzerland, and Strasbourg in France) in the trinational Upper Rhine region that provide distinct programs in preschool education, we aimed at identifying the amount of PA that is potentially possible in different educational settings. Furthermore, we analyzed PA levels in the family context to see how they are mediated by both weight and selected family habits.

## 2. Method 

### 2.1. Study Design

The study was conducted in the named four cities because these cities are all capital members in the trinational Upper Rhine region and had to be chosen due to political reasons. The study was financially supported by the Franco-German-Swiss Conference of the Upper Rhine that mandated an explorative evaluation study of different preschool settings and their impact on children's PA levels. Therefore, we only involved preschools in the city centers to avoid a town bias. All measurements were taken in the summer during three weeks, with no holiday days in the measurement time and almost similar weather conditions. The study was approved by the local Ethics Committee.

In the selected cities, we contacted the municipality of preschools which informed the principals of public preschools of our research interest. Interested preschools (Freiburg *n* = 4; Basel *n* = 5; Strasbourg *n* = 5; Landau *n* = 3) gave their consent to participation. Due to the explorative status of the study, neither a nonresponder analysis to identify patterns of nonparticipation nor a power calculation was conducted. Additionally, due to limited measurement devices, we randomly chose two preschools per city. During a parents' evening, we informed all parents with children aged five to six years about the aims of our research and invited them to enroll their children in the study. After parents had given their written informed consent for the participation of their child, *N* = 163 children were measured by direct accelerometry for five consecutive days: three weekdays (WD) and two weekend (WE) days. Parents' participation quota in the different preschools varied between 38% and 75%.

### 2.2. Measurements

Anthropometric assessment included measurement of each child's weight, height, and skinfolds. Weight status was categorized by body mass index (BMI). Children were classified as nonoverweight (<90. percentile) and overweight (>90. percentile), according to national reference BMI percentiles of German children, and the individual BMI data was converted to standard deviation scores (BMI-SDS) [19]. Skinfold thickness (SF) was determined on the right side of the body using a skinfold caliper (Lange Calipers). All measurements were done by the same investigator. To calculate the percentage body fat (%BF) from the SF, age and gender-specific regression equations were used according to Slaughter et al. [20].

By answering a questionnaire, parents provided information about their selected family markers (profession, family status, and number of children in total), their weight and height status (to calculate the BMI), their leisure time PA on weekdays and at weekends (in minutes), and their media consumption on weekdays and at weekends (in minutes). Additionally, they reported the time that their child spent in leisure time PA and screen-time entertainment (in minutes). The questionnaire used is part of the quality management of the FITOC-program (Freiburg Intervention Trial for Obese Children) and is accepted by German health insurances. Results have already been published [21].

Triaxial accelerometers (AiperMotion 440, Aipermon GmbH, Germany) were used to assess the sedentary behavior of the children, which is discussed as an independent risk factor [22]. The subjects were requested to wear the accelerometers on a belt at their hip for the whole day. Parents were asked to remove the child's accelerometer for water activities (such as swimming, taking a shower or a bath) and to refit it afterwards. They were also asked to remove it for sleeping and to refit it in the morning directly after the child got out of bed. The AiperMotion system uses 3D acceleration sensors and analyzes data with a disclosed online algorithm. The online algorithm of the AiperMotion system provides a distinction between active and passive time with a 4 s resolution, which can be used as an estimate of the time spent with and without physical activity [23]. Data from the motion sensor was exported to MATLAB (The Mathworks, USA) for further analysis. Phases without any physical activity for ≥20 min were considered as nonwear time and excluded from the calculation of the mean active time. Furthermore, days with more than 50% nonwear time in the examined period were excluded from further analysis. The ratio of active and passive time, excluding nonwear time and days with insufficient recording time, was calculated for each period (i.e., time from 9:00 a.m. to 12:00 p.m.), averaged across recording days with sufficient wear time for each subject. Subsequently, the mean activity was averaged across subjects, and the active and passive time were displayed in minutes. Although the chosen device and the measured cut points are not comparable with cut points measured by the actigraph system, the data gives reliable results within this setup.

Different schedules and curricula of the preschools in the three countries provided us with the opportunity to interpret the PA levels in the different institutions (Table 1). In France, children aged three to six years attend* l'école maternelle* in three different classes: youngest section, middle section, and oldest section. France takes seriously the education of children in their preschools as preparation for attendance at primary school. It is not “playschool”—there is a course of study that children are required to follow. The mandated curriculum leads to lessons taught during a fixed schedule for the entire preschool day (9:00 a.m.–5:00 p.m.) [24]. In Germany, different preschool programs exist (half-day or full-day). The chosen preschool settings in Freiburg and Landau provide half-day care (8:00/9:00 a.m.–1:00/2:00 p.m.), without different age classes, attended by children aged three to six years. Recommended curricula for preschools exist depending on the federal state, but only the aims of education are obligatory. Each institution is free in its creation of the schedule [25]. In Switzerland, half-day care is customary for children aged four to six years with region-specific curricula [26]. The chosen schedules in Switzerland and Germany provide high amounts of free-play time individually structured by the preschool itself. All provided timetables and further information about the playground sizes of the different institutions are presented in Tables 1 and 2.

Due to missing values in the accelerometer or questionnaire data, *N* = 54 children had to be excluded from the sample. Finally, in total *N* = 114 children (mean age = 5.3 (0.65) years) could be taken into account for the statistical analyses.

### 2.3. Statistical Analyses

All analyses were calculated with IBM^©^ SPSS^©^ Statistics Version 20. For all statistical analyses, the significance level was set at *α* = 0.05.

Preliminary analyses consisted of descriptive statistics of the anthropometrical data and family markers in the sample. One-factor ANOVAs (with Scheffe post hoc test) were used to identify mean differences in the anthropometrical data between children from different locations. Based on the parents' self-report of whether they live in a partnership or are married or if they are the only legal guardian, we classified the families as “partnership with two parents” or as “single parent.” Furthermore, we classified the families according to the number of children as “one-child families,” “two children families,” and “more than two children families.” Self-reported height and weight were used to calculate parents' BMI. To identify differences in these family markers, we calculated Pearson's chi-squared tests.

In analyzing our research question, one-factor ANOVAs (with Scheffe post hoc test) were conducted to examine differences between the children's PA in the four different cities, on weekdays as well as at the weekend. To compare full-day care with half-day care, we divided the data into two periods: morning (9:00 a.m.–12:00 p.m.) and afternoon (2:00–6:00 p.m.). In the time slot of the morning, we could be sure that all children attended preschool and for the afternoon we could be sure that only the French children attended preschool and all the other children did not. Due to this division, we can compare PA levels in the morning in the different institutional care settings and PA levels in the afternoon in preschool and in different family care settings.

A one-factor ANOVA (with Scheffe post hoc test) was calculated to examine differences between PA on weekdays and at the weekend for the total sample. Unpaired* t*-tests were used to discover mean differences in PA on weekdays and at the weekend between normal weight and overweight children. For the comparisons with the weekend, we took the time slot 9:00 a.m.–6:00 p.m.

To identify the impact of family markers (family status, number of children, media consumption, and leisure time engagement in physical activity of parents and child) and parents' weight status (BMI) on preschoolers' PA levels in the afternoon on weekdays and at weekends, we calculated a multiple stepwise regression with “time spent passive” in the concerned slot as the dependent variable.

## 3. Results

### 3.1. Description of the Sample

The sample included *N* = 114 children (*n* = 48 boys and *n* = 66 girls; mean age = 5.3 (0.65) years) from different preschools in Freiburg (*n* = 28) and Landau (*n* = 19) (Germany), Basel (*n* = 30) (Switzerland), and Strasbourg (*n* = 37)(France).  Table 3 presents the sample size and the descriptive statistics for the anthropometrical data (weight, height-SDS, BMI-SDS, and %BF) and the family markers (family status, number of children in family, and parents' BMI) differentiated by cities. In total, 82.1% of the children are of normal weight, and 17.9% are over the 90th percentile and therefore overweight. The research subjects in Strasbourg had a higher BMI-SDS (mean = 1.12 (1.78)) than all the other children at the three other locations (*P* = .00;* F* = 5.12; partial eta^2^ = 0.12). There are no significant differences in height-SDS and weight-SDS. Considering boys and girls, there were no gender differences in the anthropometrical data, except the percentage of body fat (*t* = 8.48; (*df*: 111); *P* = .00;* d* = 0.63). For mothers' and fathers' BMI, there are no significant differences between the four cities. There is no significant distribution effect for the four cities concerning family status and the number of children in the family. We also cannot state a distribution effect of overweight and normal weight children on the different family status. Even if Pearson's chi-squared test missed the set significance with *P* = .08, we would like to report that overweight children are more often found in one-child families.

### 3.2. PA in the Forenoon on Weekdays (9:00 a.m.–12:00 p.m.) Differentiated by Cities

**Figure 1:**
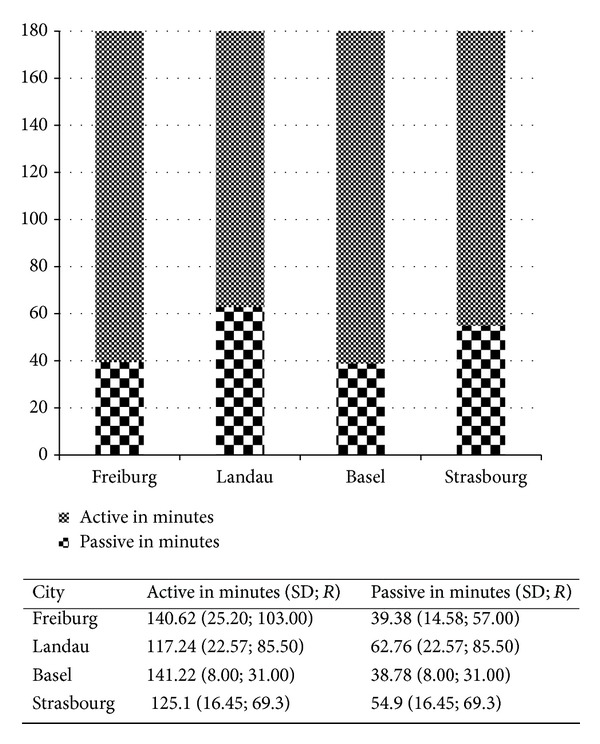
PA in the morning on weekdays (9:00 a.m.–12:00 p.m.) differentiated by countries and cities.

In the morning from 9:00 a.m. to 12:00 p.m., all of the children in the study attend preschool. Therefore, it is possible to compare how much PA the different educational systems allow in their schedules. Freiburg and Landau plan 150 minutes of unstructured play, Basel 120 minutes, and Strasbourg 30 minutes (in the form of an outdoor recess). A one-factor ANOVA showed high significant mean differences (*P* = .00;* F* = 13.01; partial eta^2^ = 0.29) between the different locations in average PA time. Scheffe's procedure shows that children in Strasbourg and Landau are significantly more passive in the morning than children in Freiburg and Basel (Figure 1). However, comparing the groups concerning their planned unstructured free-play times, we can see that children in Strasbourg are more active than in the 30-minute planned free-play time, whereas children in Landau are less active than the planned 150 minutes of unstructured free-play time.

### 3.3. PA in the Afternoon on Weekdays (2:00–6:00 p.m.) Differentiated by Cities and Family Markers

In the afternoon from 2:00 to 6:00 p.m., the studied children in Strasbourg attend preschool, whereas child care in Freiburg, Landau, and Basel is the responsibility of the family. A one-factor ANOVA did not show any significant mean differences in PA time between the children at the different locations. Scheffe's procedure did not show any significance between the groups either. To analyze the impact of different family markers (family status, number of children in the family, engagement in leisure time PA of parents and child on weekdays in minutes, media consumption of parents and child in minutes, parents' BMI, and child's BMI-SDS), we calculated a multiple stepwise regression without the French children because they are not cared for in the family context in the afternoon. The analysis showed that only the predictor “number of children in the family” has an impact on children's PA level (*P* = .04; *r* ^2^ = .11;* F* = 4.63; *β* = .33): the more siblings a child has, the more active a child is in the afternoon.

### 3.4. PA at Weekends (9:00 a.m.–6:00 p.m.) Differentiated by Cities and Family Markers

Child care at the weekend is entirely the responsibility of the family. On the one hand we tested whether there were differences in PA time between the different locations, and on the other hand we tested in an explorative way whether different family markers influence preschool children's PA level at weekends. A one-factor ANOVA for the different locations identified no significant differences in mean PA time among the children at weekends. A multiple stepwise regression showed that children's PA level at weekends is predicted by the child's BMI-SDS (*β* = .38; *P* = .01) and the mother's BMI (*β* = .32; *P* = .02) (*P* = .04; *r* ^2^ = .23;* F* = 5.92; *P* = .00).

### 3.5. PA and Weight Status

To test mean differences in PA of different weight categories, we calculated an unpaired* t*-test with all normal weight and overweight children, independent of location. Overweight children are significantly more passive on weekdays as well as at weekends (for weekdays:* t* = −2.89; (*df*: 97); *P* = .044;* d* = .21; for weekends:* t* = −2.14; (*df*: 91); *P* = .018;* d* = .29). For weekdays, a multiple stepwise regression did not provide predictors of the different PA levels of overweight and normal weight children. For the weekends, as already shown above, the child's BMI-SDS in combination with the mother's BMI is a predictor of the child's PA level at weekends.

## 4. Discussion

We found a higher percentage of overweight children in this age group, independent of location, compared to the representative German [27], Swiss [28], and French [29] reference data, but the prevalence is comparable to US data [30]. Comparing the four locations, the children in Strasbourg (France) showed a significantly higher BMI-SDS score. Our results support the literature that argues that the increasing prevalence of childhood obesity is one of the central public health challenges in modern societies [1, 31]. Evaluating PA between the weight categories, our data demonstrates a significant difference between normal weight and overweight preschoolers. Overweight children are significantly more passive on weekdays as well as at weekends. Literature provides evidence that normal weight children spend more time on average in PA than overweight children [32, 33], but there are only a few studies showing this difference in this early-age group.

As the nature of preschool has changed towards incorporating the educational domain into child care, preschool has the increasing function of teaching basic literacy and numeracy, with the aim of preparing children for school; as a result, desk-based instruction has become more important in preschools [14, 33]. With regard to the PA level, our study demonstrates that, by comparing open versus desk-based programs in the three countries, the regimented and highly structured French system leads to more inactivity in preschoolers compared to the more unstructured system in Switzerland and in Freiburg (Germany) in the morning. Desk-based care might offer fewer possibilities of PA time. Nevertheless, the French children are more active than the planned activity time in schedules. This means that the investigated French preschools probably integrate activity in their teaching.

Furthermore, the results of the second German city, Landau, show that open-orientated programs do not promote PA per se. Although the Landau and Freiburg timetables allow the same amount of free-playing time, the activity levels of children are different. It seems that open settings have to provide special structures to promote PA. Critical factors could be the number and formation of caregivers and their own engagement or training in PA. Additionally, portable equipment and larger playgrounds are associated with higher activity levels in preschoolers [34, 35]. Investigating differences in the free-play periods in Germany and Switzerland, we analyzed indoor areas, playgrounds, and outdoor possibilities and found more outdoor possibilities, as well as larger indoor areas and playgrounds in Basel and Freiburg than in Landau and Strasbourg (Table 2). With regard to the importance of indoor/outdoor play to enhance PA, the literature provides controversial results [36, 37], but Olesen et al. identified a positive association between MVPA during preschool attendance and the size of indoor area per child [38].

By collecting different family markers such as family status, number of children in the family, engagement in leisure time PA, and screen-time behavior, as well as parents' anthropometrical data, we tried to analyze in an explorative way the influence of these markers on preschool children's PA levels. For the family care in the afternoon on weekdays, we have seen that only the number of children in a family predicts the child's PA level. So, we are able to differentiate the research on families with young children as being potential risk groups. Due to our data, families with more than one (young) child seem to provide more PA than families with only one young child. These results find support in a study by McMinn et al., showing that the number of siblings, family encouragement, and family social support are associated with higher PA levels in children [39]. Our result can also be seen as important in the context of the tendency for more overweight children to come from one-child families that our data could not prove with significance, probably due to few cases in the single categories. Additionally, the multiple regression model showed that children's PA level at weekends is predicted by the child's BMI-SDS and the mother's BMI, so the more passive time spent by overweight children at weekends can be explained by their anthropometrical status. A recent study from Hesketh et al. [40] showed that PA levels in mothers and their preschool children are directly associated. They concluded that interventions targeted at mothers of young children may increase both groups' activity.

## 5. Strengths and Limitations 

Measuring PA in preschoolers is difficult due to the spontaneous and irregular type of activity in this age group. However, accelerometry is the most commonly used method for this population, but since at present the literature provides no agreement in the cut point definition for thresholds for different activity levels, the present study has the bias of not measuring with the commonly used actigraph system; so, the measured cut points are not comparable with actigraph cut points. Nevertheless, the data gives reliable results and can therefore be used to compare the different locations within this setup.

In contrast to other studies that measure PA only quantitatively or only by self-report, this study combines PA measured by objective accelerometry with the timetables of preschools and questionnaires completed by parents who gave additional information about different family markers that might influence the preschooler's PA level. Therefore, this study shows in an explorative way the effects of different preschool settings on the PA level in the forenoon of weekdays as well as the importance of the number of children in a family for the PA level on weekdays in the afternoon, as well as of the mother's and child's anthropometrical status for the child's PA level at weekends.

In addition, the presented data is limited due to several reasons. Firstly, we had only two study preschools per city. Even if we chose by random the preschools interested in the study, we would have had a selection bias. Secondly, in the studied preschools, we had to take a selection bias into account as well, because only those children whose parents were interested in the study and gave their written consent participated in the study. Thirdly, the data set only includes *N* = 114 children, so the results must be seen as explorative results.

## 6. Conclusion 

This study highlights the increasing prevalence of overweight in the preschoolers age group and the influence of multidimensional early-life factors on PA. Taking into account the high percentage of children attending a preschool, one could suggest that preschools might be a suitable setting for establishing active lifestyle habits, thereby preventing obesity. Our study has shown that “open concept” child care programs that typically feature the most free-play time seem not to promote PA per se in contrast to more desk-based programs. Therefore, preliminaries such as the special training of caregivers as well as sufficient equipment, playground size, and number of caregivers are necessary.

With regard to family context as an important early-life factor, a higher number of children in a family and the mother's and child's anthropometrical status are predictors of the engagement in PA. Further investigations into these family contexts and targeted interventions for special groups should be more focused in the future.

## Figures and Tables

**Table 1:** Timetables of preschools in the different locations.

Time	Germany (Freiburg, Landau)	Switzerland (Basel)	France (Strasbourg)
8:30–9:00	Unstructured free-play, indoor, and outdoor	Taught lesson	
9:00–9:30		Unstructured free-play, indoor, and outdoor	Taught lesson
9:30–10:00			
10:00–10:30	Breakfast	Breakfast	Breakfast
10:30–11:00	Unstructured free-play, indoor, and outdoor	Recess outdoors	Recess outdoors
11:00–11:30		Unstructured free-play, indoor, and outdoor	Taught lesson
11:30–12:00			
12:00–12:30	Sitting circle	Parents pickup	Lunch break (eating, sleeping)
12:30–1:00	Parents pickup		
1:00–1:30			
1:30–2:00			
2:00–2:30			Physical education
2:30–3:00			
3:00–3:30			Taught lesson
3:30–4:00			
4:00–4:30			Closing session (singing, unstructured play)
4:30–5:00			Parents pickup

**Table 2:** Indoor/outdoor facilities preschools.

Preschool	Indoor classrooms m^2^	Outdoor playground m^2^
Freiburg 1	48 m^2^	300 m^2^
Freiburg 2	45 m^2^	400 m^2^
Landau 1	30 m^2^	50 m^2^
Landau 2	20 m^2^	200 m^2^
Basel 1	No different classrooms, one big indoor playground 150 m^2^	300 m^2^
Basel 2	No different classrooms, one big indoor playground 180 m^2^	300 m^2^
Strasbourg 1	30 m^2^	Only 30 min. recess outdoor 200 m^2^
Strasbourg 2	35 m^2^	Only 30 min. recess outdoor 200 m^2^

**Table 3:** Distribution of the anthropometrical data and family markers in the sample differentiated by cities.

Anthropometrical data	Freiburg (D)	Landau (D)	Basel (CH)	Strasbourg (F)	Total
	M (SD)	M (SD)	M (SD)	M (SD)	M (SD)
Weight-SDS (in kg)	0.25 (0.80)	0.34 (0.86)	0.09 (0.99)	0.52 (1.03)	0.31 (0.94)
Height-SDS (in cm)	0.22 (0.91)	0.36 (0.83)	0.13 (0.92)	0.54 (1.35)	0.32 (1.06)
BMI-SDS	0.18 (0.85)	0.18 (0.78)	0.07 (1.02)	1.12 (1.78)∗	0.46 (1.33)
%BF	18.83 (4.99)	18.66 (3.63)	17.49 (4.48)	18.99 (5.21)	18.50 (4.71)
Mother's BMI	23.95 (5.04)	22.99 (3.76)	23.46 (3.77)	23.73 (4.93)	23.59 (4.46)
Father's BMI	24.90 (2.11)	25.71 (4.05)	24.92 (3.66)	25.23 (2.78)	25.15 (3.07)

Family markers	*N* (%)	*N* (%)	*N* (%)	*N* (%)	*N* (%)

Single parent	5 (17.9)	5 (26.3)	3 (13.0)	5 (16.1)	18 (17.8)
In partnership	23 (82.1)	14 (73.7)	20 (87.0)	26 (83.9)	83 (82.2)
1-child family	4 (14.3)	6 (33.3)	3 (12.0)	6 (18.2)	19 (18.3)
2-child family	13 (46.4)	8 (44.4)	12 (48.0)	15 (45.5)	48 (46.2)
<2-child family	11 (39.3)	4 (22.2)	10 (40.0)	12 (32.4)	37 (32.5)

*P ≤ .05.
